# Evaluation of a modified IDEXX method for antimicrobial resistance monitoring of extended Beta-lactamases-producing *Escherichia coli* in impacted waters near the U.S.-Mexico border

**DOI:** 10.1016/j.onehlt.2025.100997

**Published:** 2025-02-27

**Authors:** Karina Jimenez, Yuwei Kong, Yuhui Zhang, Drew Ferketic, Sana K. Nagori, Julie Yang, Anastasia A. Yulo, Brianna Kramer, Ofelia G. Prado, Taylor Cason, Renee Chowdhry, Angela Kemsley, Leopoldo Mendoza Espinosa, Joshua A. Steele, John Griffith, Jennifer A. Jay

**Affiliations:** a Department of Civil and Environmental Engineering, UCLA, Los Angeles, CA 90095, USA; b Institute of the Environment and Sustainability, UCLA, Los Angeles, CA 90095, USA; c Department of Microbiology, Immunology, and Molecular Genetics, UCLA, Los Angeles, CA 90095, USA; d Conservation Director, WILDCOAST, Del Mar, CA 92014, USA; e Instituto de Investigaciones Oceanológicas, Universidad Autónoma de Baja California, Ensenada, Baja California, Mexico; f Department of Microbiology, Southern California Coastal Water Research Project, Costa Mesa, CA, 92626, USA

**Keywords:** ESBL-EC, Tricycle protocol, Antimicrobial resistance

## Abstract

As part of a One Health approach, the World Health Organization (WHO) has deemed extended beta-lactamases-producing *Escherichia coli* (ESBL-Ec) as an appropriate proxy for antimicrobial resistance (AMR) in human, animal, and environmental samples. Traditional methods for ESBL-Ec quantification involve a labor-intensive process of membrane filtration, culturing in the presence and absence of antibiotics, and colony confirmation. The emerging modified IDEXX method utilizes IDEXX Colilert-18 test kits, recognized by the USEPA for the enumeration of total coliforms and *E. coli* in water samples, modified with cefotaxime for measurement of ESBL-Ec in environmental samples. However, this method has yet to be validated for ocean or sewage-contaminated water and has not been compared against the plate-based method with mTEC for surface water. In this study, ESBL-Ec in ocean and river waters of the Tijuana River Estuary were analyzed by three methods: membrane filtration using mTEC plates (as outlined in USEPA Method 1603), membrane filtration using TBX plates (as outlined in the WHO Tricycle Protocol), and Colilert-18 spiked with cefotaxime (Hornsby et al., 2023). Levels of ESBL-Ec were elevated in the Tijuana River Estuary and nearby ocean samples, as high as 2.2 × 10^6^ CFU/100 mL. The modified IDEXX method correlated with membrane filtration methods using selective mTEC (*r* = 0.967, *p* < 0.001, *n* = 14) and TBX (*r* = 0.95, p < 0.001, n = 14) agars. These results indicate that the modified IDEXX method can be used as a more accessible alternative to the traditional culturing methods as a screening tool for antibiotic resistance in urban aquatic environments. Advantages of the IDEXX-based method including portability, lower Biosafety Level requirements, fewer dilutions to stay within the dynamic range, greater ease of maintaining sterility during analysis, and less required staff training are discussed. Future studies into the validity of the modified IDEXX method compared to qPCR and metagenomic sequencing are needed.

## Highlights

• Elevated levels of ESBL-Ec were found at the Tijuana River Estuary and nearby ocean.
• The modified IDEXX method for ESBL-Ec correlated with membrane filtration on selective mTEC and TBX agars.
• The modified IDEXX method can be used as a monitoring tool for antibiotic resistance in ocean and sewage-impacted water.

## Graphical abstract

**Figure:**
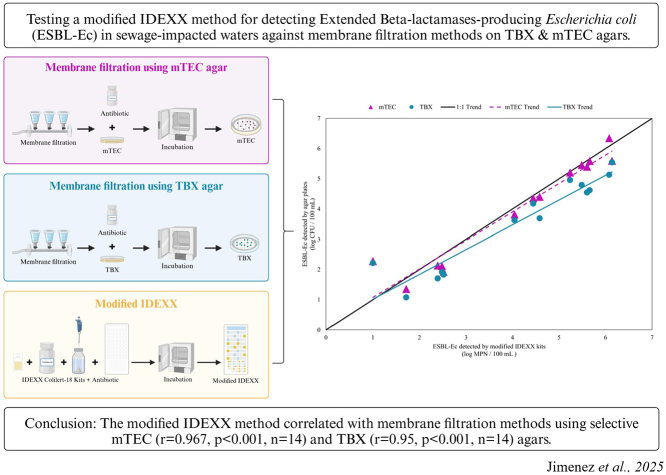

## 1 Introduction

There is a critical need for a comprehensive monitoring framework for antimicrobial resistance (AMR) monitoring in aquatic environments [[1], [2], [3], [4]]. Increasing AMR is recognized as a global health threat by the United States Environmental Protection Agency (USEPA) and the World Health Organization (WHO), reportedly responsible for 4.95 million deaths in 2019 [5]. There are pressing questions about hotspot identification, human health risk assessments, and mitigating AMR that require a standardized monitoring methodology [5].

In 2021, the World Health Organization (WHO) released the “Tricycle Protocol” for global surveillance of AMR. This protocol measures extended beta-lactamases-producing *Escherichia coli* (ESBL-Ec), an antibiotic-resistant strain of *E. coli*, as a proxy for AMR that may be present more widely throughout the bacterial community [6]. Included in the document were protocols detailing human, food chain, and environmental sample methodologies that could be implemented at laboratories on a global scale. The Tricycle Protocol provides common groundwork for a multisectoral surveillance system by outlining site selection, sampling frequency, and methods for characterizing ESBL-Ec based on country capacity. The methods range from no molecular technology up to characterization of ESBL-Ec and performing Whole Genome Sequencing (WGS). Notably, there are now additional culturing options on the market to enumerate the presence of *E. coli* in water samples that require less lab time and are more sustainable than the methods put forth by the WHO.

While the choice of ESBL-Ec for the Tricycle program is logical, and the Tricycle protocol presents a standardized watershed sampling scheme appropriate for global application, there are serious logistical issues in its implementation. First, plate-based analyses rely on spreading replicate plates with 10–100 colony-forming units per plate. For unknown samples requiring many different dilutions, the number of plates is prohibitive in terms of cost, time for spreading and counting, and incubator space. Second, the use of various broths and agars for ESBL-Ec detection globally introduces uncertainties regarding the relationship between target populations. For example, the protocols of the WHO describe culturing ESBL-Ec using *E. coli* selective media Tryptone Bile X-glucuronide (TBX) spiked with cefotaxime at a concentration of 4 μg/mL. Similarly, the United States Environmental Protection Agency (USEPA) established standardized Method 1603 for growing *E. coli* following concentration of bacteria onto membrane filters and transferring onto selective media mTEC [7]. Third, applying the Tricycle protocol often requires prohibitive costs in terms of lab time and skill. This is due to the extensive protocols required to maintain the accuracy of data in global surveillance efforts. This workflow covers plate preparation, testing of control plates, sample collection, processing, colony purification, phenotypic confirmation, *E. coli* confirmation, and isolate preservations. Addressing these implementation challenges is essential for ensuring the effectiveness and credibility of the Tricycle Protocol in environmental monitoring.

Alternatively, scientists can use ready-made kits spiked with antibiotics as a more time- and cost-effective method for quantifying ESBL-Ec in water samples. The use of the modified IDEXX method was introduced in a study conducted by Hornsby et al. in 2023. The study compared AMR *E. coli* detected from IDEXX kits supplemented with cefotaxime with the culture plating on TBX and MacConkey agars. The study included testing methods on control strains and environmental samples including surface water, surface soils, and waterfowl feces. It showed the correlation between the modified IDEXX method and plate-based methods for surface waters. While this important work provided a critical methodology for monitoring environmental antibiotic resistance in a scalable, accessible way, the performance of the method in ocean and river waters contaminated by human waste is unknown.

Additionally, mTEC is a very commonly used agar for *E. coli* detection in the U.S. and some other countries, but the relationship between the modified IDEXX and mTEC methods for detecting ESBL-Ec is unknown. As such, there are additional ways to test the modified IDEXX method to ensure its performance is comparable to the more traditional culturing methods for measuring ESBL-Ec.

Another study released in 2023 tested water and wastewater using the Tricycle Protocol along with compartment bag testing (CBT) spiked with antibiotics and found the modified CBT to be a suitable alternative to the membrane filtration methods suggested by the WHO [8]. CBT is appealing because it is portable and requires a short training period. If modified IDEXX and CBT methods were to be integrated into a global surveillance framework like the Tricycle Protocol, they would have to be performed at hotspot sources of ESBL-Ec, which the WHO outlines as waters impacted by sewage and wastewater.

To further validate and extend the modified IDEXX method presented by Hornsby et al. [9], this work aimed to:

1) independently verify its effectiveness for detecting and quantifying ESBL-Ec by comparing it to plate-based methods using both TBX and mTEC agars,
2) assess its suitability for marine and wastewater samples to identify hotspot sources, and.
3) apply it to characterize environmental AMR at a heavily impacted site near the U.S.-Mexico border prone to raw sewage contamination.

## 2 Materials and methods

### 2.1 Sample collection

#### 2.1.1 Los Angeles

The Ballona Creek Watershed encompasses 130 mile^2^ of the highly developed coastal plain of the Los Angeles Basin, with less than 20 % of the watershed being open space [10]. The flows along Ballona Creek are mostly dry weather flows and drain into the Santa Monica Bay.

**Fig. 1:**
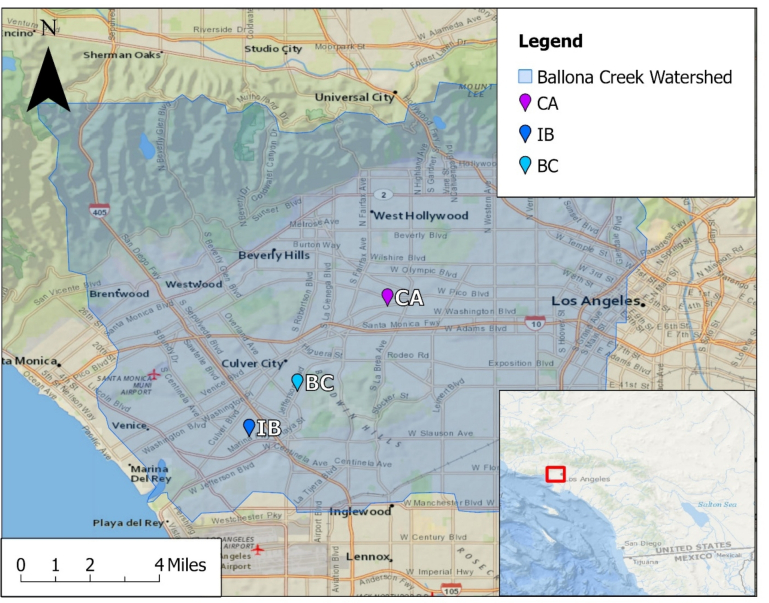
A map of sampling sites within Ballona Creek for the year 2022–2023.

A total of nine sampling campaigns were completed in the Ballona Creek Watershed during the 2022 to 2023 year. Table 1 summarizes the sampling sites, and Fig. 1 shows their location. Water sample collection occurred during the early morning hours to ensure minimal UV solar radiation. Sterile polypropylene bottles were first rinsed three times with ambient water before collection, transported on ice (4 °C), and processed within six hours of collection.

**Table 1:** Information for Ballona Creek sampling as depicted in Fig. 1.

Sampling Date	Site Name	Location	Coordinates
11/04/2022 02/13/2023 02/22/2023 03/10/2023	Cochran Avenue (CA)	Inland	34.04432, −118.35387
10/14/2022 10/21/2022 02/13/2023 02/22/2023	Inglewood Boulevard (IB)	Inland	33.98984, −118.41163
02/08/2023 02/22/2023 04/06/2023 04/18/2023	Ballona Creek (BC)	Inland	34.00874, −118.39149

#### 2.1.2 Tijuana River

The Tijuana River (TJR) is a transboundary river near the U.S.-Mexico border. The river flows from Tijuana, Mexico with a quarter of the river passing through the City of Imperial Beach, located south of San Diego, California. As Southern California's largest coastal wetland spanning 1750 mile^2^, the watershed is a unique ecosystem. Home to more than three hundred species of birds, including six endangered species, the coastal wetland is protected as a National Estuarine Research Reserve System. Under this protection, the reserve is an important site for research, water quality monitoring, education, and stewardship efforts [11]. The TJR Estuary is frequently impacted by sewage coming from Mexico. As a result of the rapid urbanization in Tijuana, the city is facing infrastructure strain. Sewage treatment plants near the border struggle to manage large inflows, especially after rainfall, leading to sewage outflows near the estuary and impacting water quality at beaches in Imperial Beach and San Diego [12].

**Fig. 2:**
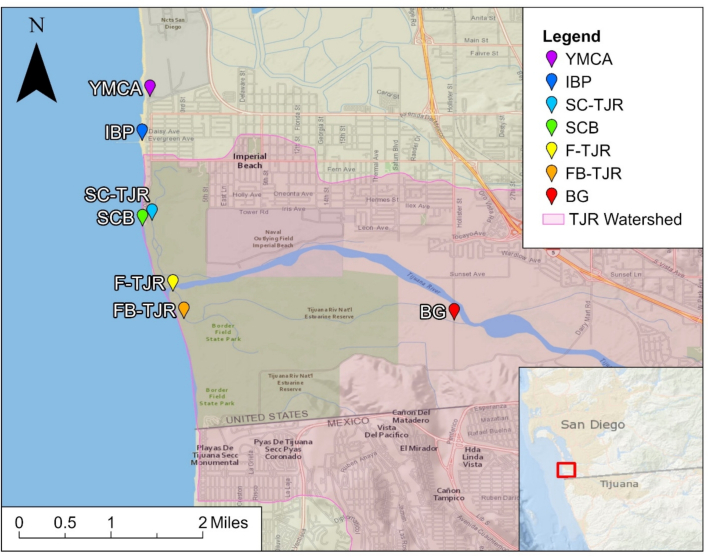
A map of the sampled sites at multiple points along the Tijuana River and Imperial Beach, located just north of the U.S.-Mexico border.

Four sampling campaigns were carried out at the TJR Watershed. Table 2 summarizes the sampling sites, and Fig. 2 shows their location. Sites were selected after consultation with WILDCOAST (COSTASALVAJE), an international community-based organization that works on coastal restoration in the TJR, and experts from the Oceanographic Research Institute of the Autonomous University of Baja California (Instituto de Investigaciones Oceanológicas, Universidad Autónoma de Baja California) to identify transboundary flows and access points along the TJR. To capture multiple points near the river outlet, samples from two inland river sites, two sites at the river mouth, and three coastal sites north of the river mouth were collected. Water sample collection occurred during the early morning hours to ensure minimal UV solar radiation. Sterile polypropylene bottles were first rinsed three times with ambient water before collection, transported on ice (4 °C) to the laboratory located at UCLA (Los Angeles, CA), and processed within six hours of collection.

**Table 2:** Sampling sites used in this study, including sampling dates, site name, coordinates, and whether the location was along the Tijuana River or the coast of Imperial Beach.

Sampling Date	Site Name	Location	Coordinates
04/14/2023 05/22/2023 09/10/2023 02/20/2024	Imperial Beach Pier (IMP)	Coastal	32.57972, −117.13314
04/14/2023 05/22/2023	Imperial Beach at Seacoast Drive (SCB)	Coastal	32.56626, −117.13309
04/14/2023 05/22/2023 09/10/2023 02/20/2024	YMCA Camp Surf (YMCA)	Coastal	32.58664, −117.13192
04/14/2023 05/22/2023 09/10/2023 02/20/2024	Tijuana River Fork (F-TJR)	River Mouth	32.55598, −117.1283
04/14/2023 05/22/2023 09/10/2023 02/20/2024	Tijuana River Mouth (FB-TJR)	River Mouth	32.55167, −117.12657
04/14/2023 05/22/2023 09/10/2023 02/20/2024	Tijuana River Southeast (BG)	River	32.55144, −117.08402
04/14/2023 02/20/2024	Tijuana River North (SC-TJR)	River	32.56713, −117.13159

### 2.2 Plate-based methods for ESBL-Ec quantification

Preparation of TBX and mTEC agars followed manufacturer suggestions, with agars poured into 60 × 15 mm Petri dishes and stored no longer than a week before use. For enumeration of ESBL-Ec, agar was spiked with cefotaxime (CTX) at 4 μg/mL after sterilization. Water samples were vacuum filtered through gridded 0.45 μm pore size mixed Ester Cellulose membrane filters. Dilutions filtered per site ranged from 0.0005 μL to 100 mL, depending on the expected bacteria concentration of the site to ensure less than 100 colony counts as recommended in the Tricycle Protocol. Duplicate plates were made for each dilution filtered. Phosphate buffer solution was used as a blank and for diluting volumes under 20 mL. Plates were sealed into bags and incubated based on manufacturer suggestions. mTEC plates were incubated at 35 °C for 2 h followed by 44.5 °C for 22 h. TBX plates were incubated at 44.5 °C for 24 h. Following incubation, plates with less than 100 colonies were recorded and stored for no longer than one week for colony purification.

### 2.3 Modified IDEXX method for ESBL-Ec enumeration

The standard methods for Colilert-18 kits were followed for *E. coli* enumeration (IDEXX Laboratories, Westbrook, ME). For the modified IDEXX method, 100 μL of 1 mg/mL cefotaxime stock solution was added to each prepared sample prior to sealing the Quanti-trays [13]. The stock concentration was determined after consideration of the pollution in the Tijuana River and consultation with Naomi Korir, a research officer with Sanivation who applied the modified IDEXX method at wastewater treatment plants in Naivasha, Kenya. A detailed protocol for the modified IDEXX method is included in Supplemental Information.

Due to the high bacterial concentration detected in preliminary sampling campaigns, samples were diluted using MilliQ purified water (Sigma-Aldrich, St. Louis, MO) up to five orders of magnitude to ensure proper enumeration by the IDEXX trays. Multiple tray dilutions were tested on each sampling day to expand the range of enumeration captured. Following incubation, IDEXX trays were inspected for fluorescent *E. coli* and ESBL-Ec. Only trays between the 1 to 2496 MPN enumeration range were recorded, as recommended by the manufacturer. Final concentrations were reported in MPN/100 mL. A detailed protocol for the modified IDEXX method is included in Supplemental Information.

### 2.4 Colony confirmation

mTEC and TBX plates with less than 100 colonies were purified on Tryptic Soy Agar (TSA) plates spiked with CTX for three of the four sampling days. After the third streak, two isolates were inoculated into Tryptic Soy Broth (TSB) and preserved in 50 % glycerol. For samples collected on February 20, 2023, isolates were transferred onto spiked TBX agar for the first streak and spiked TSA for the other two streaks. Five isolates were selected from the third streak, inoculated into TSB, and preserved in 50 % glycerol stock.

### 2.5 Species confirmation

Biochemical testing was conducted on the isolates to confirm that they were *E. coli* and not a different bacterium that can grow on mTEC or TBX plates. The ASM indole test protocol involves inoculating tubes of Tryptone broth with a purified isolate. After incubation at 35 °C for 24 h, Kovac's Reagent is added. If a red ring forms following the addition of Kovac's Reagent, the sample is positive for *E. coli*.

### 2.6 Statistical analysis

All statistical analysis was completed using Microsoft Excel. Data was filtered by two criteria: (1) No plates or trays outside of the recommended enumeration limits were used, and (2) no samples that were missing data for one of the three methods being compared. Plate counts were normalized to a 100 mL sample volume and averaged per duplicate set for final units of CFU/100 mL. IDEXX counts were converted to MPN values using the MPN chart issued with the Colilert-18 kits. MPN values were then normalized to a 100 mL sample and averaged per duplicate set for final units of MPN/100 mL.

Correlations between mTEC and TBX agar enumeration to the modified IDEXX method was calculated using the CORREL function in Excel. A two-tailed *t*-test value, *t*, was calculated based on the correlation values using the formula $t=r\sqrt{\frac{n-2}{1-r^{2}}}$ where *r* represents the correlation coefficient and *n* is the sample size. The *p*-value was then calculated using the T.DIST.2 T function in Excel with a degree of freedom *df* = *n*_*1*_ - *n*_*2*__*−*_2, where *n* represents each sampling size.

## 3 Results

### 3.1 *E. coli* and ESBL-EC in the TJR estuary and nearby coastal waters

*E. coli* and ESBL-Ec were monitored during four separate sampling campaigns at beaches just north of the U.S.-Mexico border (Fig. 2). *E. coli* results at Imperial Beach show highly elevated levels compared to water quality standards and other sites in Los Angeles, with all sites exceeding the water quality standard of 410 CFU *E. coli*/100 mL [14]. The levels of *E. coli* are highest at site BG, a freshwater site upstream of the Tijuana River Estuary, and at the southernmost beaches, sites F-TJR and FB-TJR. *E. coli* levels in the ocean tend to decrease with distance from the mouth of the estuary; notably, levels of *E. coli* at the beach site YMCA were still consistently elevated.

**Fig. 3:**
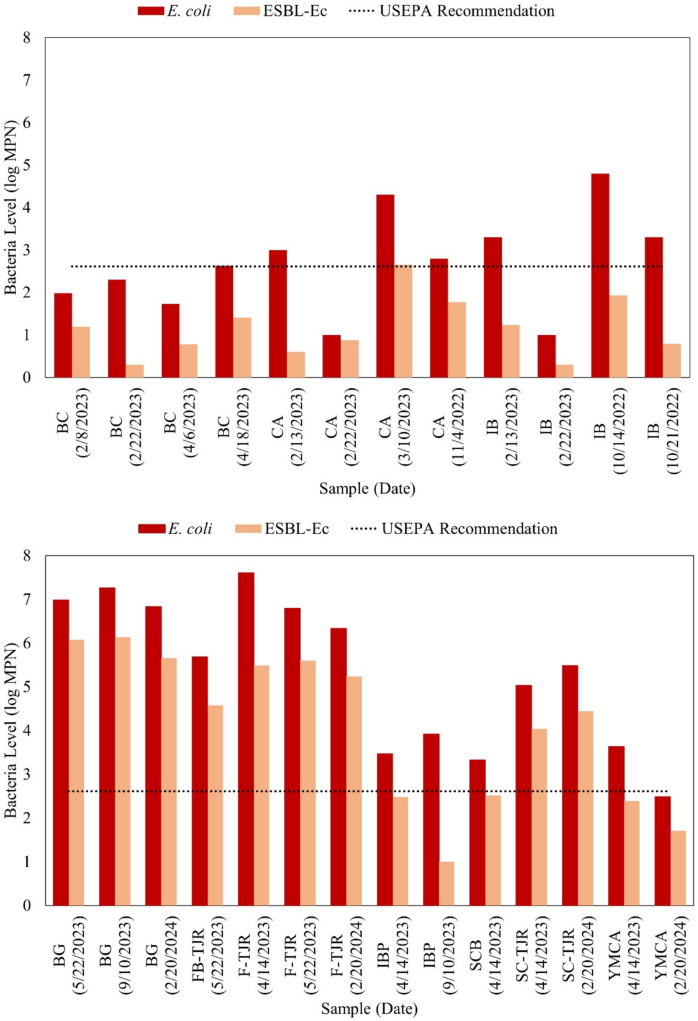
Bar graph depicting IDEXX results for detection of *E. coli* and ESBL-Ec in Los Angeles (top) and Imperial Beach (bottom). The USEPA recommendation for *E. coli* in recreational waters is 410 CFU/100 mL.

Levels of ESBL-Ec were also consistently elevated, suggesting a significant presence of AMR in waterborne bacteria. Nine of the fourteen samples from Imperial Beach exceeded the USEPA *E. coli* limit for recreational waters by at least 10 times. Sample BG 5/22/2023 showed the highest levels measured in this study at 1.2 × 10^6^ MPN ESBL-Ec/100 mL (2.2 × 10^6^ CFU ESBL-Ec/100 mL on mTEC agar), which is 10,000 times over the USEPA limit for *E. coli*. In ocean water, levels of ESBL-Ec in ocean water were highest at the sites nearest the mouth of the estuary and the border with Mexico, similar to what was observed for *E. coli*. ESBL-Ec across this study site was higher than levels observed in Los Angeles (Fig. 3). At Los Angeles, only CA 3/10/2023 slightly exceeded the USEPA standard at 457 MPN ESBL-Ec/100 mL (219 CFU ESBL-Ec/100 mL on mTEC agar).

### 3.2 Colony confirmation

The indole test was applied to 133 ESBL-Ec isolates to determine whether they were indeed *E. coli*. Only 3 colonies were negative for this assay, indicating a 97.7 % positive detection overall (See Table 3).

**Table 3:** Results from indole testing for confirmation of *E. coli* in isolates preserved from plates and IDEXX trays.

	IDEXX + CTX	mTEC + CTX	TBX + CTX
Tested	53	71	64
Positive	52	70	63
Negative	1	1	1

### 3.3 Methods comparison

**Fig. 4:**
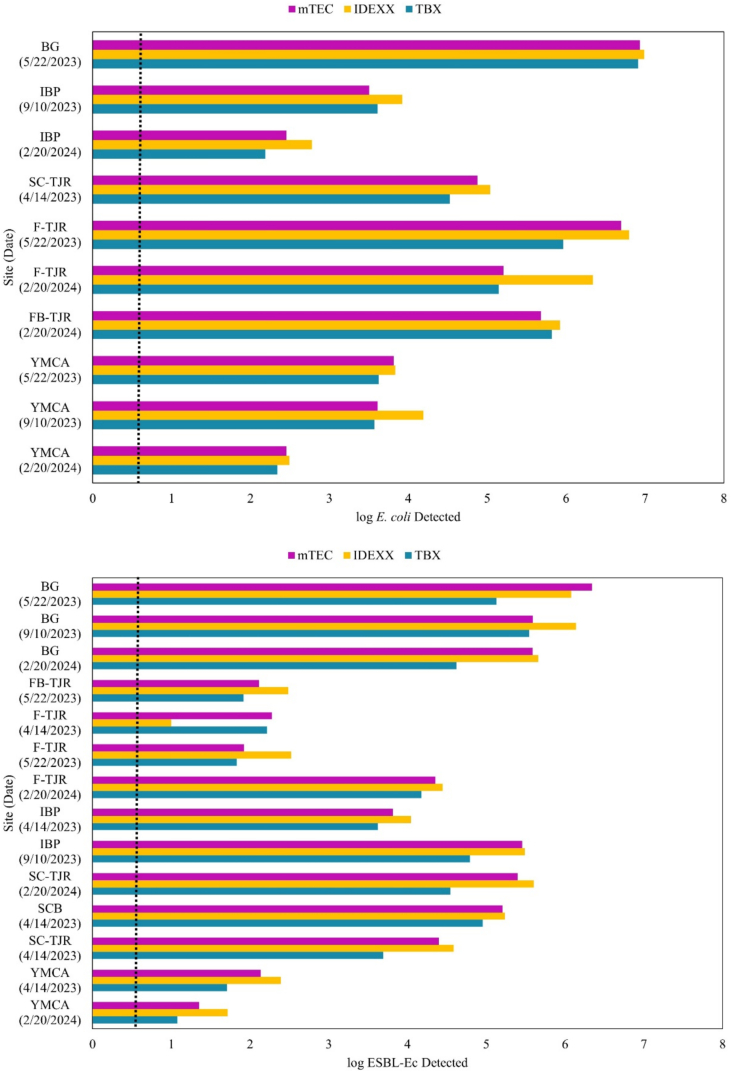
Bar graphs depicting results for detection of *E. coli* (top) and ESBL-Ec (bottom) for sites sampled at Imperial Beach. The dotted line represents the USEPA limit for *E. coli* in recreational waters. There is no current standard for ESBL-Ec.

**Fig. 5:**
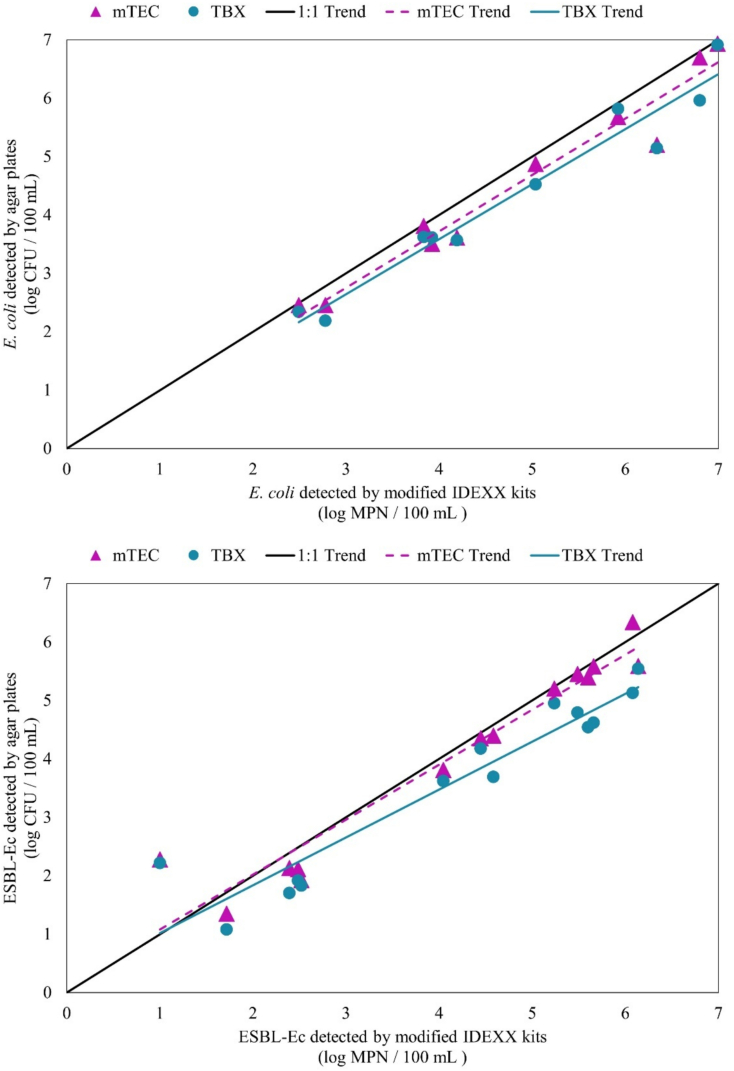
Correlation graphs for detection of *E. coli* (top) and ESBL-Ec (bottom) for samples from Imperial Beach and the Tijuana River. The modified IDEXX method correlated with membrane filtration methods using selective mTEC (r = 0.965, p < 0.001, n = 14) and TBX (r = 0.95, p < 0.001, n = 14) agars. Comparatively, the IDEXX method correlated with membrane filtration methods using selective mTEC (r = 0.978, p < 0.001, n = 10) and TBX (r = 0.975, p < 0.001, n = 10) agars.

Each water sample was analyzed for ESBL-Ec by three methods: traditional membrane filtration with mTEC agar, traditional membrane filtration with TBX, and the modified IDEXX-based method (Fig. 4). The modified IDEXX method correlated with membrane filtration methods using selective mTEC (*r* = 0.965, *p* < 0.001, *n* = 14) and TBX (*r* = 0.95, *p* < 0.001, n = 14) agars. Detection limits were similar between the methods. Comparatively, TBX, mTEC, and IDEXX were also used to measure *E. coli*. The IDEXX method correlated with membrane filtration methods using selective mTEC (*r* = 0.978, *p* < 0.001, *n* = 10) and TBX (*r* = 0.975, p < 0.001, n = 10) agars. (See Fig. 5.)

### 3.4 Cost and labor comparison

Table 4 outlines the marginal costs, which include materials for sample testing and labor associated with sample analysis. Capital costs, capital maintenance, sample collection, and transport were excluded from these calculations, as these expenses are incurred regardless of the extent of monitoring for ESBL-Ec. The estimated costs for consumable lab supplies used in the analysis were based on current market retail prices. Labor costs were based on the average salary of a laboratory technician in the United States [15]. This estimation assumes the collection of samples from 10 sites and performing 6 dilutions, as recommended by the Tricycle Protocol.

**Table 4:** Cost Comparison for 10 sites using different methods.

	Membrane filtration for TBX/ mTEC		IDEXX	
	Item	Cost	Item	Cost
Supplies	Agar	$120 for TBX $450 for mTEC	IDEXX Colilert-18 Packet	$600
	Funnels and filters	$870	Quanti-Trays	$240
	Colony confirmation	$126		
	Petri dishes	$100		
Total supply cost		$1216 (TBX) $1546 (mTEC)		$840
Labor & Time Commitment	Agar and plate preparation	2 h	Setting up IDEXX bottles and sealing	1 h
	Filtration and plating filters	5 h	Adding samples and sealing	1 h
	Counting	2 h	Counting	1 h
	Colony confirmation	8 h		
Total labor cost at $23.95 per hour		$407.15		$71.85
Total supply and labor cost	$1623.15 (TBX) $1953.15 (mTEC)		$911.85	
Total supply and labor cost per site	$162.31 (TBX) $195.31 (mTEC)		$91.18	

## 4 Discussion

Sources of contamination to the beaches in Southern San Diego County include ocean water containing partially treated and raw sewage traveling northward, as well as surface-water-based fecal pollution from the Tijuana River Estuary [12]. This work not only confirms the high levels of *E. coli* expected in this setting, but it indicates elevated levels of AMR in the aquatic microbial community.

While the detection of ESBL-Ec has been identified as a critical monitoring tool for global AMR monitoring, the traditional methods for ESBL-Ec detection are strenuous. Necessary laboratory steps include preparation of selective agar plates in the presence and absence of antibiotics, filtration of water samples at multiple dilutions, aseptically plating filters, incubating, colony counting, purification, and secondary confirmation. In contrast, IDEXX-based methods involve a five-step procedure, with the modified IDEXX method adding a sixth step of injecting antibiotics into prepared tests before sealing and incubating trays. Due to the dynamic range of IDEXX-based testing being greater than that for plate counting, fewer dilutions are necessary to achieve a reading within the readable range. Furthermore, a single tray at the proper dilution yields not only an MPN value but also a 95 % confidence interval, since the MPN is obtained through the 49 large and 48 small replicate wells on the tray.

Another advantage of the modified IDEXX-based method is the ease of transporting equipment compared to membrane filtration. In a separate study, the modified IDEXX method was piloted through a trip by air to Belize to conduct monitoring of ESBL-Ec in water (manuscript in preparation). While it was necessary to transport the IDEXX Quanti-Tray Sealer PLUS and UV light box, standard laboratory equipment such as sampling bottles, an incubator, graduated cylinders, and an autoclave for disinfection of IDEXX trays prior to disposal were present at the field site. Notably, only Biosafety Level 1 status is required for IDEXX testing due to the contained nature of the bacterial culturing whereas a Biosafety Level 2 training completion and the use of a biosafety cabinet would be needed for culturing ESBL-Ec on agar plates. Being able to analyze samples near the field location, rather than bringing the samples back to a home lab, allows for adaptive sampling during brief sampling trips [16]. Therefore, a requirement for Biosafety Level 2 status would seriously limit the locations where the method could be performed.

The Tricycle Protocol was developed by the WHO as a standardized method that would allow tracking of spatial and temporal trends in AMR nationally, regionally, and globally [6]. However, to date, only 19 countries have conducted the protocol, and only four have repeated the analysis, due to the significant implementation challenges, including the complexity of laboratory procedures and the cost of the traditional methods for ESBL-Ec detection [[17], [18], [19], [20], [21]]. The modified IDEXX method presents a more accessible, affordable, and repeatable screening tool that can be implemented into ongoing monitoring for ESBL-Ec in aquatic environments. With a simpler protocol and shorter sample processing time, such a tool can be useful for health departments and research groups intending to capture AMR trends in the environment.

In addition to the potential of the modified IDEXX method to be used as a screening tool for AMR trends, it can also be implemented into educational tools. Prevalence of Antimicrobial Resistance in the Environment (PARE), coordinated at Tufts University, is a network of educators aiming to address the lack of AMR monitoring data available by providing research opportunities for students [22]. Educational materials are provided and data provided by partnering institutions are added to a common database, after being checked for quality control. The current protocols include culture-, qPCR-, and metagenomics-based approaches for monitoring AMR. The method presented here provides an even more accessible tool for monitoring through PARE (or other networks) that could be conducted in classrooms with minimal concern about exposure to ARB.

## 5 Conclusion

Highly elevated levels of ESBL-Ec indicate concerning levels of AMR in waterborne bacteria in Southern San Diego beaches. The performance of a modified IDEXX method was shown to give comparable results to the traditional methods for ESBL-Ec analysis, validating its use for the first time in ocean and sewage-impacted water. Advantages of the modified IDEXX method for AMR monitoring include user friendliness, no requirement for Biosafety Level-2, fewer dilutions to stay within the dynamic range, greater ease of maintaining aseptic techniques, and less specialized training required for laboratory staff.

## CRediT authorship contribution statement

**Karina Jimenez:** Writing – original draft, Methodology, Investigation, Conceptualization. **Yuwei Kong:** Writing – original draft, Methodology, Investigation, Conceptualization. **Yuhui Zhang:** Writing – review & editing, Methodology, Investigation. **Drew Ferketic:** Writing – review & editing, Methodology. **Sana K. Nagori:** Writing – review & editing, Methodology. **Julie Yang:** Writing – review & editing, Methodology. **Anastasia A. Yulo:** Writing – review & editing, Methodology. **Brianna Kramer:** Writing – review & editing, Methodology. **Ofelia G. Prado:** Writing – review & editing, Methodology. **Taylor Cason:** Writing – review & editing, Methodology. **Renee Chowdhry:** Writing – review & editing, Methodology. **Angela Kemsley:** Resources, Methodology. **Leopoldo Mendoza Espinosa:** Writing – review & editing, Resources, Methodology. **Joshua A. Steele:** Writing – review & editing, Methodology. **John Griffith:** Writing – review & editing, Methodology. **Jennifer A. Jay:** Writing – review & editing, Writing – original draft, Conceptualization.

## Declaration of competing interest

The authors declare that they have no known competing financial interests or personal relationships that could have appeared to influence the work.

## Data Availability

Data will be made available on request.

## Appendix A Supplementary data

Supplementary material Image 1
