# Supplementary material for: Evaluation of a modified IDEXX method for antimicrobial resistance monitoring of extended Beta-lactamases-producing Escherichia coli in impacted waters near the U.S.-Mexico border
Source: One Health. 2025 Feb 27;20:100997. doi: 10.1016/j.onehlt.2025.100997 (PMC11930104; doi:10.1016/j.onehlt.2025.100997)
Supplement: Supplementary file 1 — Supplementary material [file mmc1.docx]

**Supplemental Information**


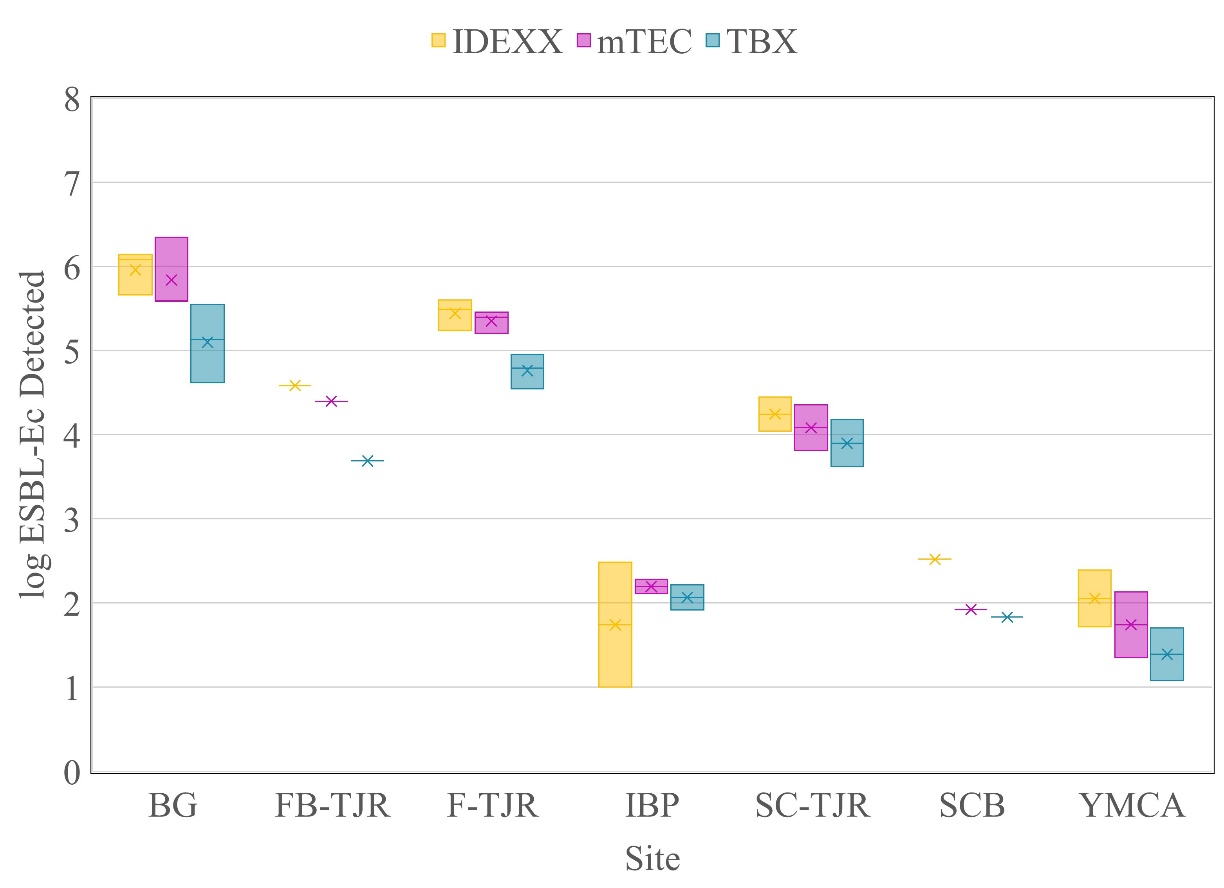


***Figure S1.*** *Box and Whisker Plots for E. coli (top) and ESBL-Ec (bottom) detected across each site.*

**Modified IDEXX Colilert-18 SOP**

*Jimenez et al., 2025*

**Equipment**

- IDEXX Quanti-Tray Sealer
- 365-nm UV light
- Autoclave
- Incubator

**Reagents**

- IDEXX Colilert-18 test kits
- Cefotaxime salt

**Preparation**

1. Sanitize work station.
2. Gather supplies:
   1. Samples (stored below 10°C for less than 6 hours)
   2. Sterile bottles (at least 100 mL in volume)
   3. Sterile graduated cylinders
   4. Pipette (At least one that can transfer 100 μL)
   5. Pipette tips
   6. IDEXX Quanti-Trays
   7. IDEXX Colilert-18 powder packets
   8. Baskets (To hold unsealed IDEXX trays upright)
   9. Sterile water
   10. Cefotaxime (CTX) salt (stored at 4°C)
   11. DI water
   12. 0.2 μM filter
   13. 5 mL Syringe (1)
   14. 1.7 mL microcentrifuge tubes
3. Turn on the Quanti-Tray Sealer.
4. Set incubator at 35°C.
5. Prepare CTX Solution (1 mg/mL):
   1. Suspend 0.01 g (10 mg) of Cefotaxime salt in 10 mL of deionized water.
   2. Filter-sterilize using 0.2 μM filter with 5 mL syringe.
   3. Transfer 500 μL of the filter-sterilized concentrate to a sterile 1.7 mL microcentrifuge tube.
   4. Label the tubes with the drug name, concentration, and date.
   5. Freeze all aliquots at -20°C for later use or use immediately.
   6. Thaw aliquots at room temperature and cover from direct light prior to use. For this protocol, once an aliquot is thawed and re-frozen twice, the remaining concentrate should be discarded.

**Procedure**

1. Label the sterile bottles and IDEXX Quanti-Trays (Site-Date-Test-Dilution).
2. Add sterile water needed for dilution into the sterile bottles. Shake sample bottles, let it settle for a few seconds, and add raw sample needed to complete dilution.
3. Add the IDEXX Colilert powder packets to the bottles.
4. Shake bottles until the reagents dissolve.
5. Repeat step 5 for each site.
6. Shake each bottle and add 100 μL of CTX solution.
7. Repeat step 7 for each site.
8. Pour the contents of each bottle into the designated Quanti-Tray and keep upright.
9. Carefully place a Quanti-Tray into the Quanti-Tray rubber insert.
10. Insert the Quanti-Tray rubber insert into the Quanti-Tray Sealer.
11. Repeat steps 10-11 for each site.
12. Incubate Colilert trays laid flat with seal up at 35°C ± 0.5°C for 18 - 22 hours
13. Record the time when you began incubating.
14. Turn off the Quanti-Tray Sealer and sanitize work station.

**ESBL-E. coli Counts**

1. Once incubation is complete, sanitize work station.
2. Take a Quanti-Tray, draw a dash on all the yellow squares, and record total coliforms.

$$ESBL Total Coliform= \frac{Yellow big squares}{Yellow small squares}$$

3. Repeat step 2 for each site.
4. Turn UV box on.
5. Insert a Quanti-Tray into the UV box, draw a perpendicular dash on all the fluorescent squares, and record ESBL-producing E. coli.

$$ESBL producing E. coli= \frac{Flourescent big squares}{Flourescent small squares}$$

6. Repeat step 5 for each site.
7. Record data.
8. Turn off UV box, and sanitize work station,
9. Dispose of trays with positive wells as biohazard.

**Sanitizing IDEXX Trays**

Autoclave any trays with ESBL-Ec positives in an autoclave-safe biohazard bag. If an autoclave is not available, heating the tray to 70°C or boiling will kill the bacteria inside the wells (Spinks et al., 2006).

**Determining Dilutions**

A preliminary sampling campaign is recommended when first applying the methods to a new area. This will help gauge what bacterial concentrations are expected and will allow a narrower range of dilutions that can be performed with each sampling. However, if preliminary sampling is not an option, doing a six-fold 1:10 dilution series will greatly increase the enumeration range.

**For more information and a video of the unmodified procedures visit:**

https://www.idexx.com/en/water/water-products-services/colilert-18/

**References**

Colilert-18 product insert. Colilert-18-06-02027-27.pdf

Korir, N., Kirby, A., Murphy, J., & Berendes, D. (2021). Evaluation of Prevalence and Changes in Antimicrobial-Resistant Faecal Organisms in Faecal Sludge and Wastewater Treatment Plants, Naivasha, Kenya. *The 6th International Faecal Sludge Management Conference (Virtual).*

Spinks AT, Dunstan RH, Harrison T, Coombes P, Kuczera G. Thermal inactivation of water-borne pathogenic and indicator bacteria at sub-boiling temperatures. Water Res. 2006 Mar;40(6):1326-32. doi: 10.1016/j.watres.2006.01.032. PMID: 16524613.
